# High‐energy visible light at ambient doses and intensities induces oxidative stress of skin—Protective effects of the antioxidant and Nrf2 inducer Licochalcone A in vitro and in vivo

**DOI:** 10.1111/phpp.12523

**Published:** 2019-11-17

**Authors:** Tobias Mann, Kerstin Eggers, Frank Rippke, Mirko Tesch, Anette Buerger, Maxim E. Darvin, Sabine Schanzer, Martina C. Meinke, Jürgen Lademann, Ludger Kolbe

**Affiliations:** ^1^ Beiersdorf AG, Research and Development Hamburg Germany; ^2^ Center of Experimental and Applied Cutaneous Physiology Department of Dermatology, Venerology and Allergology Charité – Universitätsmedizin Berlin Berlin Germany

**Keywords:** antioxidant, licochalcone A, reactive oxygen species, sunscreen, visible light

## Abstract

**Background:**

Solar radiation causes skin damage through the generation of reactive oxygen species (ROS). While UV filters effectively reduce UV‐induced ROS, they cannot prevent VIS‐induced (400‐760 nm) oxidative stress. Therefore, potent antioxidants are needed as additives to sunscreen products.

**Methods:**

We investigated VIS‐induced ROS formation and the photoprotective effects of the Nrf2 inducer Licochalcone A (LicA).

**Results:**

Visible spectrum of 400‐500 nm dose‐dependently induced ROS in cultured human fibroblasts at doses equivalent to 1 hour of sunshine on a sunny summer day (150 J/cm^2^). A pretreatment for 24 hours with 1 µmol/L LicA reduced ROS formation to the level of unirradiated cells while UV filters alone were ineffective, even at SPF50+. In vivo, topical treatment with a LicA‐containing SPF50 + formulation significantly prevented the depletion of intradermal carotenoids by VIS irradiation while SPF50 + control did not protect.

**Conclusion:**

LicA may be a useful additive antioxidant for sunscreens.

## 1 INTRODUCTION

Life on earth is strongly depending on solar radiation. However, solar radiation can also cause severe damage as a function of intensity and wavelength. In the last decades, photobiological research focused on the deleterious effects of ultraviolet (UV) light with the highest photon energy and the biggest impact on skin although the UV waveband accounts for only 5% of the solar spectrum.^1^ It is well established that cellular damage, in particular by UVA, is mediated through the generation of reactive oxygen species (ROS) in sun‐exposed skin. At the opposite end of the solar spectrum, investigations on the effects of infrared (IR) light have resulted in conflicting effects and conclusions 2, 3, 4, 5; physiological doses are mostly deemed non‐hazardous^6^, ^7^ and even may be beneficial.^8^ The intermediate visible spectrum (VIS 400‐760 nm) was long regarded harmless although more than 50% of solar radiation reaching the surface of the earth is visible light with the global spectral irradiance at a latitude of 45°N at solar noon peaking at around 500 nm.^9^ Kielbassa et al,^10^ on the other hand, had reported oxidative DNA damage induced by short‐wave VIS (400‐450 nm) in Chinese hamster cells already in 1997.

In contrast to dermatology, the clinical relevance of high‐energy visible light (HEVIS) induced ROS effects on ocular tissues, in particular the retina, has been acknowledged much earlier, prompting questions regarding potentially harmful effects on sun‐exposed skin.^11^, ^12^ Recently, UV/VIS (385‐405 nm) was reported to induce delayed CPD formation in vivo.^13^ In contrast to other wavelengths of VIS, HEVIS exposure leads to a significant decrease in viability of different skin cell lines and more pronounced shrinkage of the extracellular matrix (ECM).^14^, ^15^, ^16^ Like UVA, HEVIS appears to exert its effects mainly through the generation of ROS, accounting for a substantial part of the amount generated by natural midday sunlight in human skin.^17^, ^18^ Blue light photon ROS production efficacy corresponds to 25% of UVA in human keratinocyte mitochondria.^19^ ROS detoxification in skin is achieved by low molecular weight antioxidants, such as Vitamins C and E, and carotenoids like β‐carotene, as well as by enzymes and antioxidant proteins, many under the control of nuclear factor erythroid 2‐related factor 2 (Nrf2), the master regulator of cellular redox signaling and antioxidant defenses.^20^, ^21^ Carotenoids are photoprotective, lipophilic plant‐derived pigments with highest concentration in the superficial stratum corneum (SC) and aggregation at skin surface.^22^, ^23^ Carotenoids mainly exhibit absorbance maximum at wavelengths in the range of visible light 21 and are rapidly degraded by blue light radiation in human skin ex vivo 24 and in vivo, indicating ROS formation.^25^ However, endogenous restoration takes up to 24 hours.^25^ Furthermore, blue light exposure can activate Nrf2 as a protective endogenous response, in human epidermal cells.^26^, ^27^ Loss of Nrf2 has negative consequences for skin homeostasis, repair, and disease, suggesting that further activation is beneficial also for augmented skin photoprotection.^20^, ^28^, ^29^

Hence, HEVIS leads to a ROS‐induced imbalance between protective and aggressive factors, resulting in tissue damage, permanent pigment darkening, photodermatoses,^30^, ^31^, ^32^, ^33^ melasma,^34^, ^35^ and skin aging.^19^, ^36^ Accordingly, HEVIS, but not red light, induces hyperpigmentation, in particular in subjects with more pigmented skin.^37^, ^38^, ^39^, ^40^ Furthermore, VIS (400‐700 nm) and UVA1 (340‐400 nm) synergistically induce skin pigmentation and erythema.^41^, ^42^ Based on these effects, photoprotection against VIS/HEVIS is increasingly advocated.^40^, ^41^, ^42^, ^43^, ^44^, ^45^, ^46^, ^47^ Diffey and Osterwalder^48^ even postulated that the labelled sunscreen sun protection factor (SPFs) may overestimate protection in natural sunlight due to the greater spectral output in the visible region compared with UV solar simulation, contributing 17% to an erythemal reaction. Adding VIS‐absorbing mineral filters to sunscreens significantly improves protection against the development of VIS‐induced hyperpigmentations.^49^, ^50^ However, inclusion of such mineral pigments, for example, iron oxide and non‐nano‐titanium dioxide, results in tinted formulations,^45^, ^49^, ^50^ limiting their broad application. Recent evidence suggests that plant‐derived antioxidants can protect VIS‐exposed skin from ROS‐induced oxidative stress,^51^, ^52^, ^53^ but their effects on Nrf2 remain to be established. A number of botanical ingredients including bixin, salidroside, tanshinones, caffeic acid, ferulic acid, quercetin, rutin, and the algae‐derived mycosporine‐like amino acids (MAAs) shinorine and porphyra‐334 were shown to mitigate UV‐induced cell damage via upregulation of Nrf2,^20^, ^28^, ^29^, ^54^, ^55^, ^56^, ^57^ but little is known about their protective effects against VIS. Licochalcone A (LicA) extracted from the roots of *Glycyrrhiza inflata* was identified as very potent antioxidant, inhibiting of UV‐induced ROS generation, and activator of Nrf2 in primary human fibroblasts.^58^, ^59^, ^60^ LicA stimulated the Nrf2/ARE signaling pathway by factor 9 at 2 µmol/L concentration.^61^ Here, we present data on the effect of VIS on cutaneous oxidative stress levels at doses and intensities representing one hour of sun exposure in summer in Central Europe. Furthermore, we present results that show the protective effect of LicA on VIS‐induced oxidative stress and Nrf2 induction in vitro, and as protectant against SC carotenoid degradation in vivo.

## 2 MATERIAL AND METHODS

### 2.1 Active ingredients

Licorice extract from the roots of *Glycyrrhiza inflata* contained 21% LicA and was purchased from Beijing Gingko. For cell culture experiments, a solution of the LicA‐rich licorice extract in DMSO was prepared and diluted with DMEM, and the final DMSO concentration in culture was 0.1%. The sunscreen (in vivo SPF 50+/in vitro UVA‐PF 40) applied in the in vitro and in vivo studies was an oil in water emulsion containing 0.025% licorice extract, corresponding to 0.005% LicA (for ingredients according to INCI see Appendix S1).

### 2.2 Light sources

Various light sources with different filters were used to irradiate cells in vitro and skin in vivo. All doses are given as physical, not erythemally weighted doses, since the erythema inducing potential of the various spectra is quite different. For detailed information, see Appendix S1, Table S1 and Figure S1, S2, S3, and S4. In order to compare the spectral output of the light sources with the ambient sunlight, the solar spectrum in Hamburg, Germany, was measured on a sunny and a cloudy summer day on top of a building free of any shadowing. Every few minutes, the radiometer (Spectro 320D, Instrument Systems) recorded a spectrum in the range of 280‐1700 nm with 1 nm steps.

### 2.3 In vitro studies

#### 2.3.1 Absorption spectra of sunscreen formulations used in the studies

**Figure 1:**
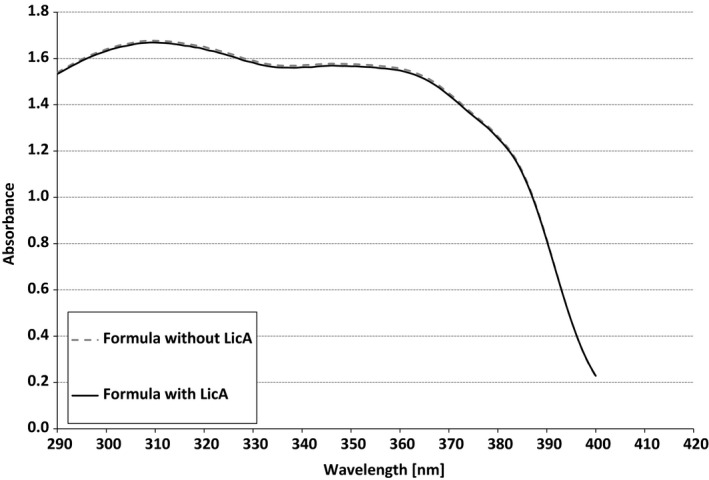
Absorption spectra of the SPF 50 + sunscreen products with or without LicA used in this study

The absorption spectra of sunscreen formulations (Figure 1) were determined following the methodology described in the ISO24443 for the determination of UVA protection. An UV/VIS Spectrometer Lambda 650 S (PerkinElmer) equipped with 150 mm integrating sphere was used for the measurement.

#### 2.3.2 Cell culture

Primary human dermal fibroblasts of Caucasian donors, phototypes I to III, were isolated from skin biopsies derived from plastic surgery in healthy donors as described elsewhere.^62^ Cells were cultured in Dulbecco's modified Eagle's medium (Invitrogen) supplemented with 10% fetal calf serum (FCS; PAA), penicillin/streptomycin 50 µg/mL, and 1% L‐glutamine (all from Invitrogen) at 37°C and 7% CO_2_. Details of the Nrf2 activation assay are provided as Appendix S1.

#### 2.3.3 Irradiation experiments

In order to assess the potency and wavelength dependency of VIS irradiation to induce ROS formation in vitro, cultured primary human fibroblasts were washed with PBS and irradiated with VIS at wavelengths of ≥400 nm, ≥450 nm, ≥500 nm, and ≥585 nm, respectively. The overall ROS levels after irritation were measured applying a modified DCF H2DCFDA (Life Technologies GmbH) method, which is not selective for single ROS species,^63^ at a final concentration of 10 µmol/L after 20 minutes of incubation. Measurements were carried out with a plate reader (Infinity 1000 M Pro, Tecan) at an excitation wavelength of 492 nm and an emission wavelength of 520 nm.

### 2.4 In vivo study

Based on the results of the in vitro experiments, a double‐blind, vehicle‐controlled in vivo study was conducted to determine the protective effect of the sunscreen with and without LicA against VIS‐induced depletion of cutaneous carotenoids. The study was performed in accordance with the Good Clinical Practices and the principles of the Declaration of Helsinki and approved by the local Ethics Committee of Charite, Berlin (EA1/228/17). Skin carotenoids were measured noninvasively on three test areas on the inner forearm as baseline value using resonance Raman spectroscopy with an excitation wavelength at 488 nm.^22^, ^25^, ^64^ On two of the areas, 2 mg/cm^2^ of the sunscreens was applied, respectively, whereas the third area served as untreated positive control. The total test area was immediately irradiated with VIS (Skintrek^®^ PT3, blueVIS mode: 410‐600 nm, maximum at 440 nm, 100 J/cm^2^) for 42 minutes. Immediately after completion of irradiation, the skin carotenoids were determined again as described above. For more details, see Appendix S1.

### 2.5 Statistics

The in vitro data were analyzed using the *t* test function of Microsoft Excel. A *P*‐value <.05 was regarded as statistically significant. For the statistical analysis of the in vivo study the nonparametric Wilcoxon test, SPSS Statistics 19 was used. A *P*‐value <.05 was regarded as statistically significant.

## 3 Results

### 3.1 In vitro studies

**Figure 2:**
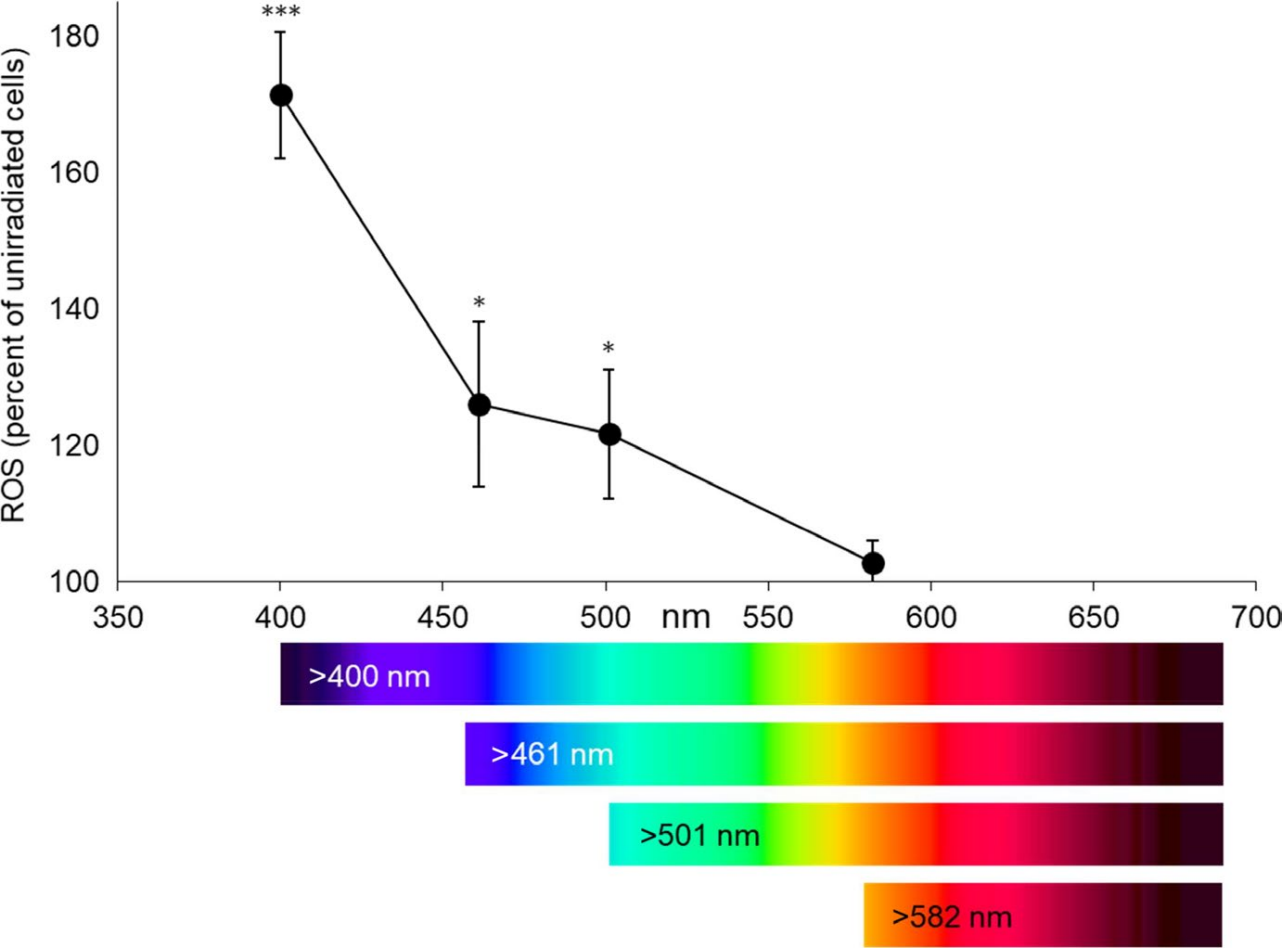
Reactive oxygen species formation in cultured human fibroblasts depending on wavelength. Cultured cells were exposed to 150 J/cm^2^ of visible light >400 nm, >450 nm, >500 nm, and >585 nm, respectively. The Oriel 1600 W Solar Simulator filtered for VIS irradiation was used for the experiments. Results of unirradiated cells were set to 100%; n = 9; mean ± SD. Significant differences were marked (*P < .05, ***P < .001)

The conditions for our in vitro experiments were based on measurements of solar spectral radiation from extraterrestic satellite data^1^ and incident radiation measurements in Hamburg, Germany, on a sunny and a cloudy summer day. They showed that a dose of 150 J/cm^2^ VIS, as used in the in vitro experiments, can be acquired within 1 hour (Table 1). Initial experiments with different VIS wavelengths revealed that HEVIS ranging from 400 to 450 nm resulted in the highest ROS levels in cultured human fibroblasts. With increasing wavelengths, there was a continous decrease in ROS generation, with only minor effects with VIS >500 nm (Figure 2).

**Table 1:** Solar irradiances in Hamburg in summer on a cloudy and a sunny day. Averaged data calculated from various spectra measured between 10 am and 2 pm on the days indicated

	Cloudy weather (Hamburg, June 18, 2010)	Sunny weather (Hamburg, June 17, 2010)
VIS irradiance (mW/cm^2^)	13	43
Time to acquire 150 J/cm^2^ VIS (min)	192	58
UVB irradiance (mW/cm^2^)	0.06	0.19
Time to acquire 150 mJ/cm^2^ UVB (min)	41	13
UVA irradiance (mW/cm^2^)	1.5	4.3
Time to aquire 2.5 J/cm^2^ UVA (min)	28	10
IRA irradiance (mW/cm^2^)	7	25
Time to acquire 600 J/cm^2^ (min)	1445	400

**Figure 3:**
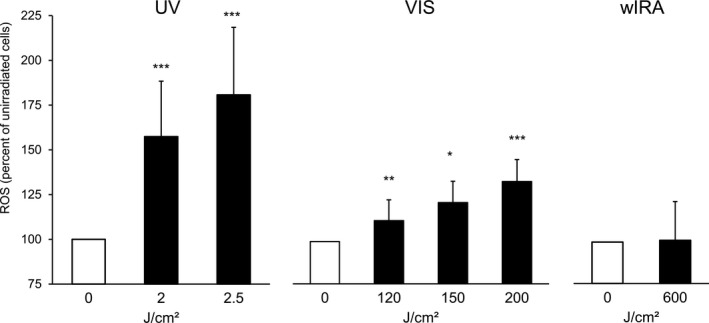
Formation of ROS in cultured human fibroblasts induced by solar simulated UV, VIS, and water‐filtered IRA. Cells were irradiated with various doses of UV and VIS and a fixed dose of water‐filtered IRA. Results of unirradiated cells were set to 100%; n = 7 for UV and VIS; n = 8 for wIRA; mean ± SD. Significant differences were marked (*P < .05, **P < .01, ***P < .001). An Oriel 1600 W Solar Simulator filtered for UV irradiation, another Oriel 1600 W Solar Simulator filtered for VIS irradiation, and a Hydrosun wIRA 505 for IRA irradiation were used

Irradiation of cultured fibroblasts with increasing doses of solar simulated UV radiation (UVA + UVB) and VIS ≥400 nm (Figure 3) resulted in a dose‐dependent increase of ROS formation as compared to unirradiated controls. Note that the time to acquire 2.5 J/cm^2^ solar UV radiation is about 10 minutes sun exposure in Hamburg in June at noontime and clear sky (Table 1). With water‐filtered IRA, we could not induce any ROS, even with 600 J/cm^2^ in cultured fibroblasts under tightly controlled temperature conditions. The irradiation with 600 J/cm^2^ corresponds to almost 7 hours of sun exposure at noontime and clear skies, and we even applied the doses at irradiance five times higher than the real sun under clear sky.

**Figure 4:**
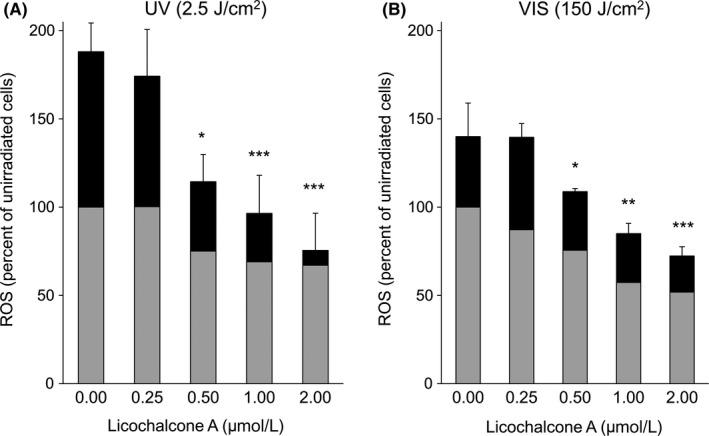
Reactive oxygen species formation in irradiated cultured human fibroblasts after pretreatment with LicA. After incubation with LicA at various concentrations, cells were exposed either to (A) UV (2.5 J/cm^2^) or (B) VIS >400 nm, 150 J/cm^2^. An Oriel 1600 W Solar Simulator filtered for UV irradiation and another Oriel 1600 W Solar Simulator filtered for VIS irradiation were used. Gray bars show oxidative stress levels without irradiation, and black bars represent the additional oxidative stress induced by irradiation. Results of unirriadiated and untreated cells were set to 100%; n = 7; mean ± SD. Significant differences were marked (*P < .05, **P < .01, ***P < .001)

**Figure 5:**
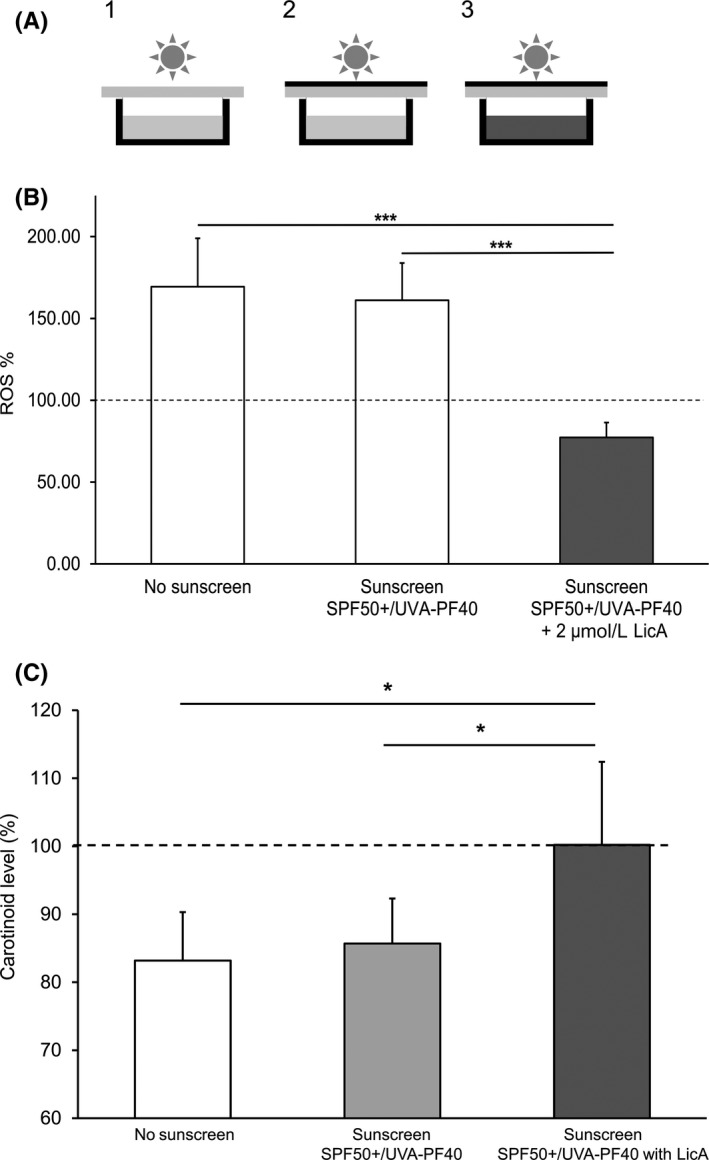
A, Setup for the irradiation of cultured cells under sunscreen protection. During irradiation, culture plates were covered by PMMA plates without^1^ or with^2^, ^3^ sunscreen SPF 50+/ UVA‐PF 40 containing LicA applied. To mimic penetration of antioxidant into the skin, some cultures were incubated with LicA prior to irradiation.^3^ B, ROS formation after VIS irradiation with SPF 50+/ UVA‐PF 40 sunscreen protection and with or without LicA. Fibroblast cultures were irradiated with 150 J/cm^2^ VIS through a PMMA plate (white bar), a PMMA plate covered with sunscreen SPF 50+/UVA‐PF 40 (gray bar) or a PMMA plate with the same sunscreen applied and additionally 2 µmol/L LicA in the culture medium (black bar), respectively. Significant differences were marked (***P < .001). An Oriel 1600 W Solar Simulator filtered for VIS irradiation was used. C, Mean carotenoid levels after irradiation relative to initial values measured in vivo in the skin. Prior to irradiation with blue light (100 J/cm^2^), for the spectrum of the Skintrek^®^ PT3 filtered for UV irradiation see Figure S3 in Appendix S1, skin areas were either left untreated (white bar), or protected by a sunscreen (SPF 50+, UVA‐PF 40) containg only UV filters (gray bars) or the same sunscreen containing additionally LicA (black bar). Carotenoids in skin were measured in vivo by resonance Raman spectroscopy (n = 10; mean ± SD; *P < .05)

Treatment of the cell cultures with sulforaphane and LicA revealed an approximately five times more potent Nrf2 activation by LicA (see Figure S8, Appendix S1). Hence, instead of sulforaphane, LicA served as high control in all further experiments with additional antioxidants. Pretreatment with LicA at concentrations of 0.25 to 2.0 µmol/L for 24 hours markedly reduced VIS‐ and UV‐induced ROS formation in a dose‐dependent manner as compared to untreated controls (Figure 4). A concentration of 1 µmol/L LicA reduced the VIS‐induced ROS formation to, or even below, the level observed in unirradiated cells, whereas 0.25 µmol/L LicA had only a minor effect. With 2 µmol/L LicA, ROS formation was almost halved as compared to no pretreatment either with or without VIS exposure. In contrast, vitamin E provided a comparable reduction of VIS‐induced ROS formation only at a concentration of 100 µmol/L (see Figure S9, Appendix S1). In cultures exposed to UV (2.5 J/cm^2^), 2 µmol/L LicA reduced ROS generation to the level of unirradiated cells (Figure 4A). When cell cultures were protected only by a PMMA plate covered with the sunscreen SPF 50+/UVA‐PF 40 with LicA (Figure 5A), this had no effect on VIS‐induced ROS formation (Figure 5B). Hence, neither UV filters nor LicA had any VIS‐filtering effect. However, when 2 µmol/L LicA was added to the culture medium, ROS levels were reduced below those obtained with unirradiated cells. Various other antioxidants were also tested but did not provide any Nrf2 induction and protection from ROS formation at comparable concentrations (see Figure S6 and Figure S7, Appendix S1).

### 3.2 In vivo study

The basic carotenoid levels before irradiation and treatment in the test areas (control, sunscreen without and with LicA) were 2.83 ± 0.24, 2.76 ± 0.21, and 2.66 ± 0.19 arbitrary units (a.u.), respectively. Exposure to VIS (maximum at 440 nm) at a dose of 100 J/cm^2^ led to a significant reduction of carotenoids in the control area to 2.38 ± 0.19 a.u. and in the skin area treated with sunscreen without LicA to 2.37 ± 0.19 a.u. immediately after completion of irradiation, corresponding to approximately 85% of the baseline values (*P* < .01). The cutaneous carotenoids were preserved (2.66 ± 0.21 a.u.) in the skin area treated with the LicA‐containing sunscreen. The relative changes in skin carotenoids to initial values were significantly different between both sunscreen formulations and between the sunscreen with LicA and the control area (*P* < .05; Figure 5C). Skin temperature showed only a modest increase by 1°C at average during irradiation.

## 4 DISCUSSION

Our data confirm that ROS are dose‐dependently generated in human dermal fibroblasts in vitro by irradiation with VIS and UV, respectively, and that the highest ROS yields are obtained with the shortest VIS wavelengths, that is, with blue‐violet light (HEVIS). They further demonstrate that (a) the pretreatment of fibroblasts with the antioxidant LicA decreased ROS formation induced by 150 J/cm^2^ VIS or 2.5 J/cm^2^ UV to the level in unirradiated cells or even below, (b) conventional UV filters, designed to protect against UV radiation, are ineffective in reducing VIS‐induced ROS formation, (c) the protective efficacy of LicA against VIS‐induced ROS formation and VIS‐induced carotenoid depletion does not result from filtering effects, and (d) a physiological dose of 150 J/cm^2^ VIS associated with a significant amount of ROS generation can be acquired within approx. 1 hour on a sunny summer day in Hamburg, Northern Germany. The latter observation confirms the relevance of the irradiation dose used in the in vitro experiments.

The in vivo experiment shows a significant depletion of cutaneous carotenoids after VIS irradiation (100 J/cm^2^) of skin treated with a sunscreen (SPF 50+/UVA‐PF 40) without LicA, similar to unprotected skin as corrobotated by earlier findings.^24^, ^25^ The SC carotenoid content remains completely preserved after treatment with the same sunscreen containing LicA. As epidermal carotenoids are concentrated in the superficial SC layers,^22^, ^23^ protection from VIS‐induced depletion can be achieved already by short‐term application of the antioxidant LicA. β‐Carotene degradation by VIS irradiation (400‐800 nm, 24.6 J/cm^2^) of human skin biopsies was reduced by 22% by pretreatment with a formulation containing 3% niacinamide; addition of 0.5% dl‐α tocopherol yielded protection by 65%, which was attributed to the antioxidant function of the vitamins.^24^ As suppression of lipid peroxidation was demonstrated for LicA in several biological systems^65^ and is well established also for carotenoids,^21^ we postulate this mode of action also for the instant carotenoid stabilizing effect of LicA found in our in vivo study. Since the effector phase of Nrf2 activation is considered time‐dependent, its involvement in the reactions of the viable skin layers remains to be elucidated in future studies. However, earlier data showed that a reduction of UV‐induced erythema was evident 5 hours after application of LicA, proving rapid cutaneous penetration and efficacy.^58^

The development of sunscreens initially focused on protection from UV as the main cause of skin photodamage. Recent findings on the negative effects of UV/Visible radiation on the skin including delayed CPD formation^12^, ^13^, ^18^ underline an important role of antioxidants in photoprotection. Vitamin E has been applied in sunscreens for decades, and a recent study provided evidence that it effectively inhibits DNA damage by UVA1‐induced photosensitization reactions^66^ Furthermore, ROS formation in retinal pigmented epithelial cells upon exposure to blue light and tobacco smoke toxins was significantly reduced by incubation with Vitamin E.^67^ However, these effects occured at much higher concentration (100 and 10 µmol/L, resp.) than analyzed in our studies (1‐2 µmol/L, Figure S6 and S7, Appendix S1) and as corroborated by our experiments with Vitamin E at high dosages (Appendix S1, Figure S9). At lower concentrations, Vitamin E (2 µmol/L) and Vitamin C (14 µmol/L) did not protect human fibroblasts from UVA1‐induced heme oxygenase 1 mRNA expression.^68^ Vitamin C provided antioxidative effects at a concentration of 1 mmol/L in mouse embryonic fibroblasts after riboflavin‐activated UVA1 irradiation^69^ However, preincubation with 1 mmol/L Vitamin C did not inhibit blue light‐induced cytotoxicity in human keratinocytes.^14^ Significant and meaningful Nrf2 activation by Vitamin C was recently revealed in primary skin fibroblasts only at concentrations above 70 µmol/L.^70^ Hence, the low antioxidant activity found in our studies may be attributable to the experimental setup with application of actives in low concentrations. The addition of antioxidants of plant origin, mostly polyphenols like flavonoids, to sunscreen formulations is now commonly advocated.^36^, ^71^ They protect against oxidative stress by scavenging free radicals emerging, for example, from porphyrins and carotenoids sensitized by the residual solar radiation that passes the organic and inorganic filters. Thus, they form a second line of defense below the layer of these filters.^59^, ^72^ A UVA/UVB sunscreen provided protection from VIS‐induced ROS, IL‐1a, or MMP‐1 release in human skin equivalents only after addition of an antioxidant combination of feverfew extract, soy extract, and gamma tocopherol.^51^ Single application of a cream containing 1.5% *Hypericum perforatum* extract, panthenol, tocopherol acetate, and allantoin reduced VIS/NIR radiation‐induced ROS production by 80% whereas the vehicle cream yielded a reduction by 60%.^52^ This difference was enlarged by prolonged application of the creams for 4 weeks.^53^ Our investigations provide evidence for VIS‐protective effects of a specific, well‐defined LicA‐containing plant extract at much lower concentration (0.025%). Other plant‐derived antioxidants like tanshinone (5 µmol/L), quercetin (7.5 µmol/L), caffeic acid (15 µmol/L), salidroside (20 µmol/L), bixin (20 µmol/L), and the MAAs shinorine and porphyra‐334 (100 µmol/L) also induce Nrf2,^29^, ^54^, ^55^, ^56^, ^57^ however, at higher concentrations than LicA (1µmol/L). This highlights the importance of Nrf2 activation for the reduction of VIS‐induced oxidative stress. Although prolonged and strong activation of Nrf2 may have adverse effects on skin, especially in UVR‐induced carcinogenesis, sustained activation of Nrf2 appears to suppress the harmful effects of chronic UVR exposure.^73^ Hence, limited pharmacological Nrf2 activation was suggested as a promising strategy for cancer chemoprevention.^20^ LicA and the well‐known Nrf2 activator sulforaphane were reported to induce the nuclear translocation of Nrf2 at a similar level^60^; however, our data obtained with a different assay indicate higher efficacy of LicA. Both phytochemicals provide anticancer properties against a variety of tumors^74^, ^75^ including, for LicA, oral squamous cell carcinoma and skin papilloma.^74^ Licorice and licorice extracts/derivatives are Generally Recognized as Safe (GRAS) for use in foods in the USA by the Food and Drug Administration (FDA) (21 CFR 184.1408). In conclusion, the transient activation of Nrf2 by LicA and other phytochemicals is considered beneficial and safe.

A growing body of evidence suggests complex effects of blue light on skin cells.^14^, ^15^, ^16^, ^19^ Blue light at different wavelengths induces varying degrees of oxidative stress, which may involve carbonylated proteins acting as photosensitizers.^76^ Whereas blue light is cytotoxic for keratinocytes, dermal fibroblasts and skin‐derived endothelial cells at short wavelengths (410‐420 nm), and high fluences, it inhibits cell proliferation and induces differentiation at lower fluences and/or higher wavelengths (up to 480 nm) dose‐dependently, presumably by photolytic release of NO.^14^, ^77^ Furthermore, papillary and reticular fibroblasts respond differently to high‐dose blue light, for example, with regard to their metabolic activity.^78^ Accordingly, an opsin receptor (peropsin) sensitive for blue‐violet light was identified on human keratinocytes^79^ and fibroblasts,^80^ and opsin receptor expression was disrupted by blue light irradiation along with an oxidative stress response.^81^ In line with these findings, Regazzetti et al 82 identified opsin 3 as the key sensor in melanocytes responsible for hyperpigmentation induced by blue light (reviewed by 47). Dendritic cells (DC) are also affected by blue light irradiation, which in vitro impairs DC maturation upon activation and subsequent allogeneic stimulatory capacity.^83^ Such findings further support recent recommendations to extend sun protection “beyond UV radiation”.^36^, ^71^ However, our experiments show that the relevance of nonthermal IR at intensities representing natural solar radiation is neglectible with respect to ROS formation in cultured human fibroblasts, as corroborated by others.^3^, ^6^

Efficient photoprotection including HEVIS is essential for light‐skinned individuals as well as individuals with darker skin complexion who respond to VIS radiation with a more intense and sustained pigmentation than to UVA1.^37^ Furthermore, patients with various skin diseases with or without dyspigmentations require photoprotection extending to the VIS spectral range. Thus, melasma relapses can occur despite the use of effective UV sunscreens,^34^, ^35^ and consequently, additional HEVIS protection has yielded beneficial effects in this condition.^45^, ^84^ Photoprotection beyond UV may also be beneficial in patients with photodermatoses triggered by VIS including polymorphous light eruption,^30^, ^31^ chronic actinic dermatitis,^31^ and solar urticaria.^32^, ^33^ In patients with porphyrias, opaque sunscreens blocking blue‐violet visible light are effective but conventional formulations filtering only UV rays are rarely helpful.^85^ Furthermore, carotenoids may be helpful in the prevention and treatment of some photodermatoses.^31^ Hence, stabilization of epidermal carotenoid levels upon ambient blue light exposure may benefit affected patients. More in vivo randomized controlled trials are also needed to further establish the efficacy of topical antioxidants against VIS‐induced skin hyperpigmentation.^47^

High‐energy visible light phototherapy is applied in the treatment of a number of benign and malignant skin disorders including acne, atopic dermatitis, psoriasis, actinic keratosis, and basal cell carcinoma.^86^ For phototherapy of eczema, UV‐free blue light is effective, with local hyperpigmentation as the only side effect.^87^ However, the numerous and partly contradictory effects on skin cells depending on dose and wavelength must be considered, especially as the clinical relevance and interdependence of the biological responses are not yet fully understood.^88^

The limitations of the presented studies are twofold: First, we showed that the Nrf2 inducer LicA more potently reduced ROS at lower concentrations than other antioxidants. However, since our in vivo study showed LicA efficacy already 42 minutes after application, this experimental setup does not allow to reveal Nrf2‐dependent effects since these would take more time to develop. Thus, demonstration of a superior antioxidant efficacy due to Nrf2 induction is a mandatory objective for future studies. Yet, this would require a very different study design. Second, we did not investigate potential contributions of other components of the *Glycyrrhiza inflata* root extract. LicA was identified as the dominant antioxidant and Nrf2 inducer in *Glycyrrhiza inflata* root extract,^61^ and the extract used in our studies was enriched for LicA content from around 3% to 21%. Although it was shown before that the anti‐inflammatory activity of this extract is due to the LicA content,^58^ the involvement of other components in skin protection from HEVIS damage remains to be clarified.

In summary, based on the fact that also wavelengths beyond the UV spectrum, in particular HEVIS light, are capable to induce oxidative stress leading to skin damage, it appears logical to develop novel sunscreens by inclusion of antioxidants.^36^, ^46^, ^51^, ^52^, ^53^, ^59^, ^71^, ^72^ LicA, a retrochalcone derived from *Glycyrrhiza inflata*, exerts strong antioxidant and Nrf2 activating efficacy in VIS‐irradiated human fibroblasts and protective effect on cutaneous carotenoids in vivo. The contribution of Nrf2 activation to the in vivo protection from HEVIS‐induced oxidative stress remains to be elucidated in further studies.

## CONFLICTS OF INTEREST

TM, KE, FR, MT, AB, and LK are employees of Beiersdorf AG, and none of the other authors has a conflict of interest to declare.
