# Supplementary material for: High‐energy visible light at ambient doses and intensities induces oxidative stress of skin—Protective effects of the antioxidant and Nrf2 inducer Licochalcone A in vitro and in vivo
Source: Photodermatol Photoimmunol Photomed. 2019 Nov 17;36(2):135–44. doi: 10.1111/phpp.12523 (PMC7078816; doi:10.1111/phpp.12523)
Supplement: Supplementary file 1 [file PHPP-36-135-s001.docx]

**Supporting Information**

High energy visible light at ambient doses and intensities induces oxidative stress of skin – Protective effects of the antioxidant and Nrf2 inducer Licochalcone A in vitro and in vivo

Mann, T.^1^, Eggers, K.^1^, Rippke, F.^1^, Tesch, M.^1^, Buerger, A.^1^, Darvin, M.E.^2^, Schanzer, S.^2^, Meinke, M.^2^, Lademann, J.^2^, Kolbe, L.^1^

^1^Beiersdorf AG, Research and Development, Hamburg, Germany

^2^Center of Experimental and Applied Cutaneous Physiology, Department of Dermatology,

Venerology and Allergology, Charité – Universitätsmedizin Berlin, Berlin, Germany

**Appendix S1**

**Ingredients of Sunscreen Lotion SPF 50+, UVA-PF 40 (INCI):**

Aqua, Alcohol Denat, C12-15 Alkyl Benzoate, Octyldodecanol, Butyl Methoxydibenzoylmethane, Octocrylene, Titanium Dioxide (nano), Bis-Ethylhexyloxyphenol Methoxyphenyl Triazine, Butylene Glycol Dicaprylate/Dicaprate, Cetyl Alcohol, Sodium Phenylbenzimidazole Sulfonate, Cetearyl Alcohol, Myristyl Myristate, Tocopheryl Acetate, Glycyrrhiza Inflata Root Extract, Glycerin, Diethylhexyl Butamido Triazone, Ethylhexyl Methoxycinnamate, Tapioca Starch, Glyceryl Stearate SE, Xanthan Gum, PEG-40 Castor Oil, Sodium Cetearyl Sulfate, Acrylates/C10-30 Alkyl Acrylate Crosspolymer, Trimethoxycaprylylsilane, Trisodium EDTA, Phenoxyethanol, Methylparaben, Parfum

**Cell culture experiments**

For each wavelength, 6 samples were analysed. The experiment was repeated with different doses (120, 150, and 200 J/cm^2^) of VIS ≥ 400 nm (n = 7) and solar simulated UV radiation (2 J/cm^2^ and 2.5 J/cm^2^) (n = 7). Unirradiated cell cultures in PBS served as control.

In another set of *in vitro* irradiation experiments the effect of LicA on ROS generation by cultured dermal fibroblasts was investigated. Cultured primary human fibroblasts were incubated in medium supplemented with different concentrations (0.25, 0.5, 1.0, and 2.0 μM) of LicA for 24 hours. Subsequently, the cell cultures were washed with PBS to remove the LicA containing media and irradiated in PBS with 150 J/cm^2^ visible light (VIS) with wavelengths of ≥ 400 nm or solar simulated UV radiation (2.5 J/cm²), respectively. Untreated and unirradiated cell cultures served as control.

In a further experiment, the cultured fibroblasts were exposed to VIS ≥ 400 nm (150 J/cm^2^) filtered by a sunscreen (SPF50+/ UVA-PF40) with LicA applied to a plexiglass plate fixed above the cell cultures. Subsequently, ROS levels with and without irradiation were determined by a modified DCF assay as described above.

**Nrf2 activation assay**

The KeratinoSens^TM^ cell line (1) was used to determine the Nrf2-inducing potential of anti-oxidants. Cells were incubated with the test substances for 24 hours. The substances were solved in DMSO, the final concentration of DMSO did not exceed 1%. After incubation, the cells were washed, the luciferase reaction was started and measured as described in the publication (1).

(1) Andres E, Sá-Rocha VM, Barrichello C, Haupt T, Ellis G, Natsch A. The sensitivity of the KeratinoSens™ assay to evaluate plant extracts: a pilot study. *Toxicol In Vitro.* 2013;27:1220-5.

***In vivo* study**

Ten healthy volunteers aged between 24 and 56 years with skin type II or III were included in the study. The subjects were not allowed to apply any cream to the test areas within 72 hours prior to the study and had to take a shower or bath not later than 4 hours before the study started. On the inner forearm of the volunteers, three areas of 5 cm^2^ each were marked. A time of 42 minutes with power adjusted to 40 mW/cm^2^ was needed to achieve a VIS dose of 100 J/cm^2^ (HBM-1 power meter, Hydrosun Medizintechnik GmbH, Type, Müllheim, Germany). The irradiated area was cooled by a fan and the skin temperature controlled by a non-contact thermometer (Rytek Schlender Messtechnik, Rüthnick, Germany). In order to investigate a possible interaction of LicA with the Raman spectrum of carotenoids, Raman measurements (excitation wavelength 532 nm) were conducted with LicA and the UV filters applied in both sunscreens. Whereas LicA presented a minor Raman signal at 1525 cm^-1^, the UV filter Bis-Ethylhexyloxyphenol Methoxyphenyl Triazine exhibited a much more intensive peak (fig. S5). The concentration of this UV filter in the sunscreens was 800x higher than LicA; however, carotenoid levels of skin protected with the sunscreen without LicA were similar to unprotected skin. Furthermore, LicA does not absorb light at a wavelength of 488 nm (2) and, thus, was not excited by resonance Raman spectroscopy as applied in the *in vivo* study. Hence, an interaction between LicA and the carotenoid measurements was excluded.

(2) Monti S, Manet I, Manoli F, Marconi G. Structure and properties of licochalcone A–human serum albumin complexes in solution: a spectroscopic, photophysical and computational approach to understand drug–protein interaction. *Phys Chem Chem Phys* 2008;10:6597–6606.

**Table S1: Irradiation devices**

| Oriel 1600 W Solar Simulator filtered for VIS irradiation used in the *in vitro* studies | |
| --- | --- |
| Spectralrange | Filters |
| ≥ 400 – 750 nm | Dichroic filter reflecting 420 – 630 nm from New Port 400FH90-50S cut-on Filter from L.O.T.  PGO‑57400 IRA-Filter from L.O.T. |
| ≥ 461 – 750 nm | Dichroic filter reflecting 420 – 630 nm from New Port 450FH90-50S cut-on Filter from L.O.T.  PGO‑57400 IRA-Filter from L.O.T. |
| ≥ 501 – 750 nm | Dichroic filter reflecting 420 – 630 nm from New Port 500FH90-50S cut-on Filter from L.O.T.  PGO‑57400 IRA-Filter from L.O.T. |
| ≥ 582 – 750 nm | Dichroic filter reflecting 420 – 630 nm from New Port 585FH90-50S cut-on Filter from L.O.T.  PGO‑57400 IRA-Filter from L.O.T. |

| Oriel 1600 W Solar Simulator filtered for UV irradiation used in the *in vitro* studies | |
| --- | --- |
| Spectralrange | Used filters |
| ≥ 290 – 400 nm | Dichroic filter reflecting 280 – 400 nm from New Port WG320 from Schott |

| Hydrosun wIRA 505 for IRA irradiation used in the *in vitro* studies | |
| --- | --- |
| Spectralrange | Used filters |
| ≥ 765 – 1360 nm | HYD-RG780-99-5X3PT from Schott |

| Skintrek® PT3 filtered for UV irradiation used in the *in vivo* studies | |
| --- | --- |
| Spectralrange | Used filters |
| ≥ 410 – 600 nm | blueVIS mode |


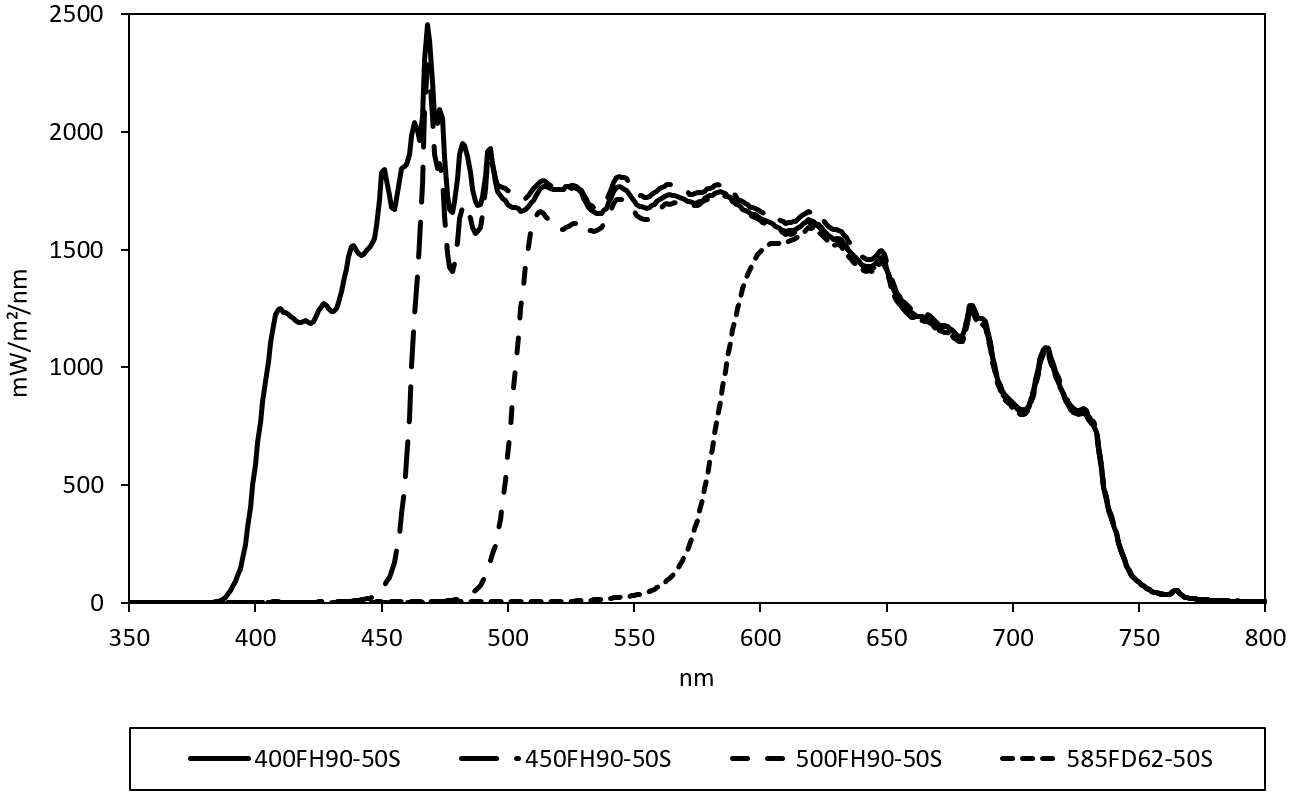


**Figure S1:** Spectra of the Oriel 1600 W Solar Simulator after filtering with additional suitable filters (all L.O.T-QuantumDesign GmbH, Darmstadt, Germany) for *in vitro* irradiation with VIS in specific spectral wavelength ranges.


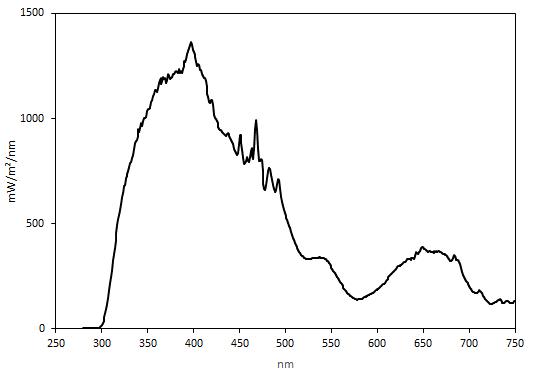


**Figure S2:**, Spectrum of the Oriel solar simulator equipped with a dichroic filter reflecting in the UV (280-400 nm) and a WG320 filter (all L.O.T-QuantumDesign GmbH, Darmstadt, Germany) for UV irradiation.


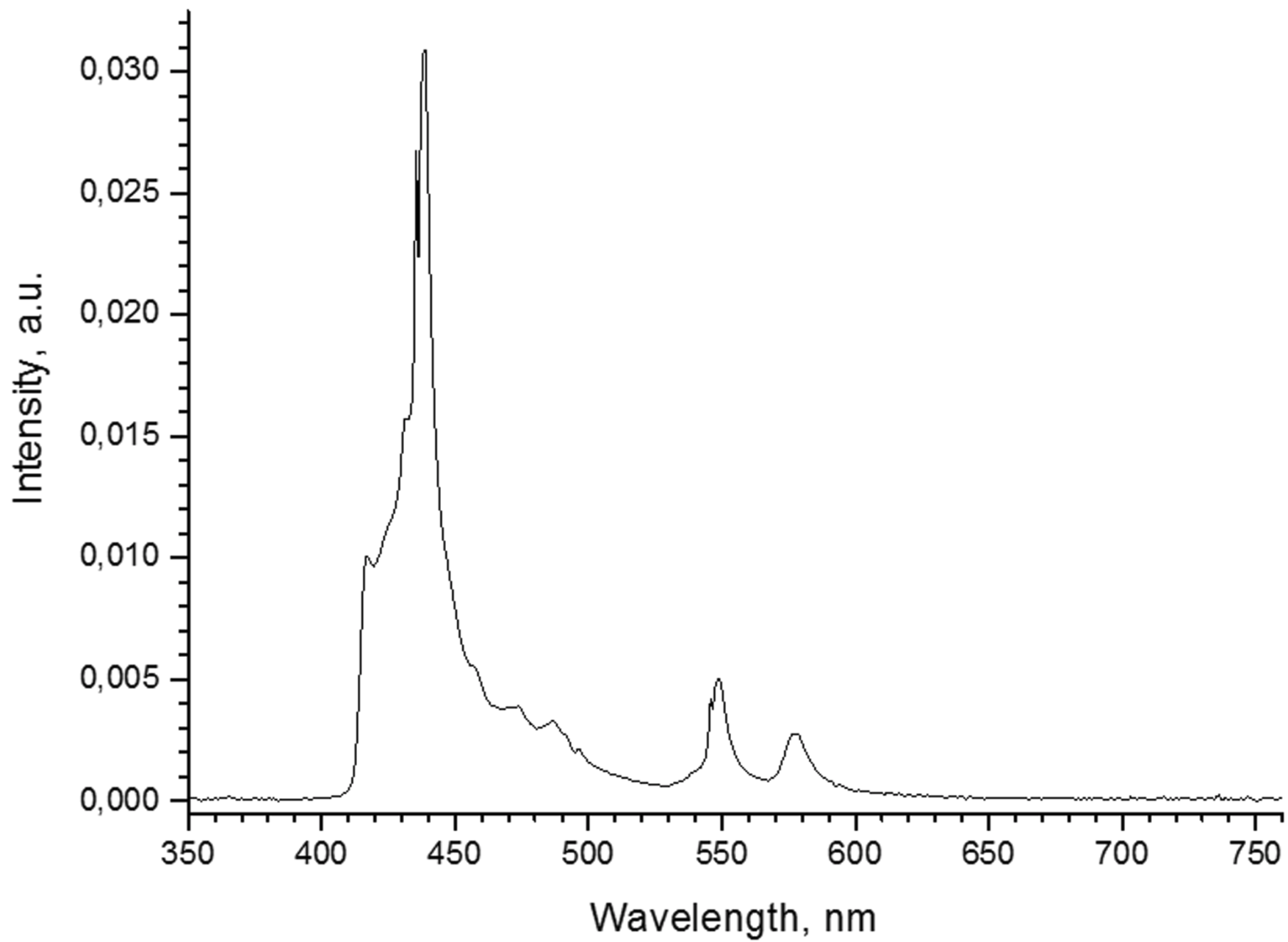


**Figure S3:** Spectrum of the Skintrek PT3 used for *in vivo* irradiation.

**Figure S4:** Spectrum of the Hydrosun wIRA 505. Infrared radiation of cell cultures was performed with a Hydrosun wIRA 505 (Hydrosun Medizintechnik GmbH, Type HBM-1, Müllheim, Germany).


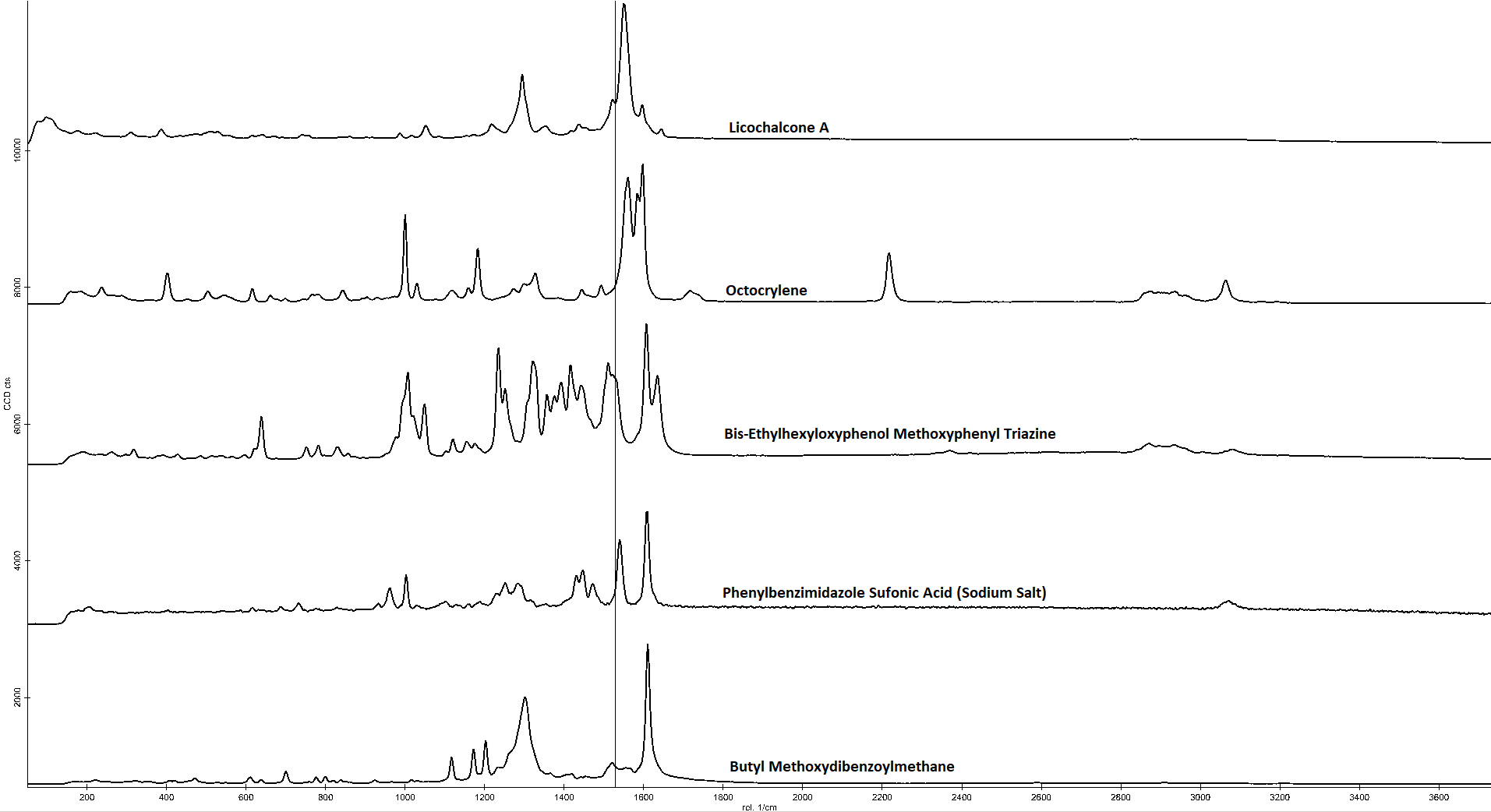


**Figure S5:** Raman spectra of LicA, Octocrylene, Bis-Ethylhexyloxyphenol Methoxyphenyl Triazine, Phenylbenzimidazole Sulfonic Acid (Sodium Salt), and Buthyl Methoxydibenzoylmethane (alpha500, WITec, Ulm, Germany).


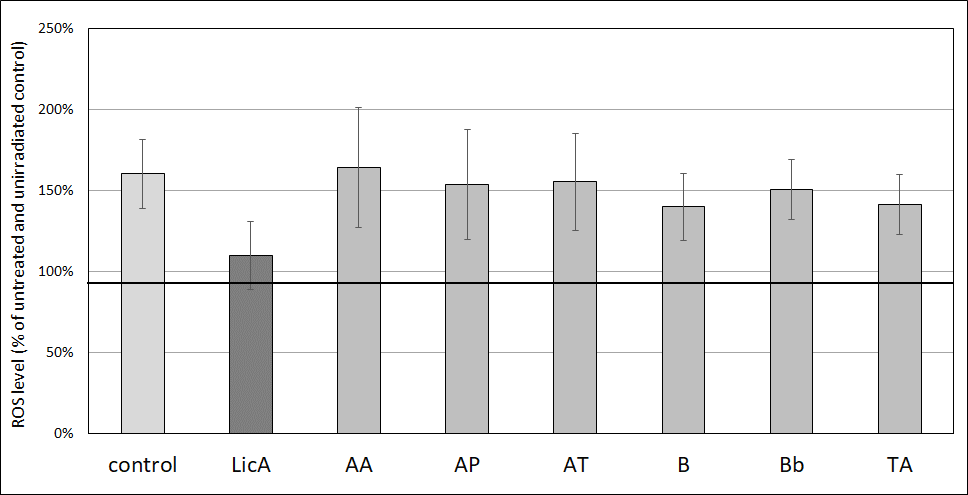


**Figure S6:** Effect of various antioxidants on ROS formation induced by 150 J/cm^2^ VIS. The level of ROS in untreated fibroblasts was set to 100 %. Cells were incubated with antioxidants at 2 µM for 24 hours. LicA = Licochalcone A, AA = ascorbic acid, AP = ascorbyl palmitate, AT = ascorbyl tetraisopalmitate, B = baicaline, Bb= bisabolol, TA = tocopheryl acetate. Significant difference as marked, ***p≤ 0.001.


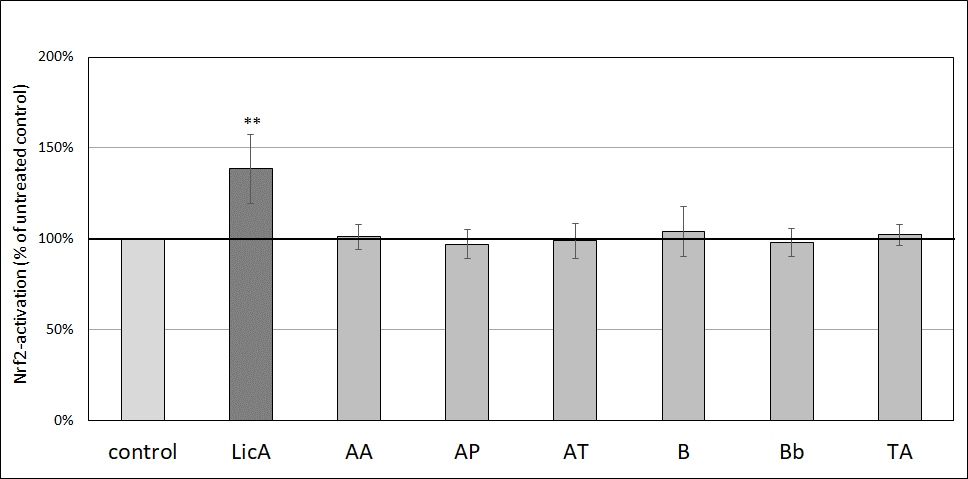


**Figure S7:** Effect of various antioxidants on Nrf2 activation. The level of Nrf2 activity in untreated cells was set to 100 %. Cells were incubated with antioxidants at 1 µM for 24 hours. LicA = Licochalcone A, AA = ascorbic acid, AP = ascorbyl palmitate, AT = ascorbyl tetraisopalmitate, B = baicaline, Bb= bisabolol, TA = tocopheryl acetate. Significant difference as marked, **p≤ 0.01.


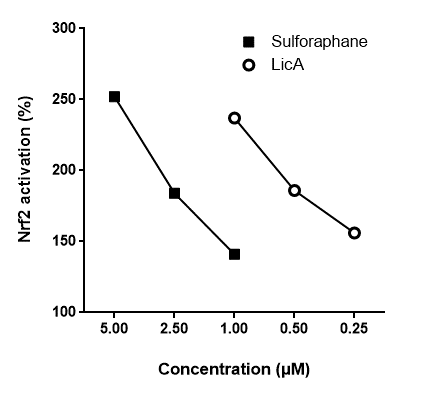


**Figure S8**: Nrf2 activation by Sulforaphane and LicA. The level of Nrf2 activity in untreated cells was set to 100 %. Cells were incubated with anti-oxidants at various concentrations for 18 hours.


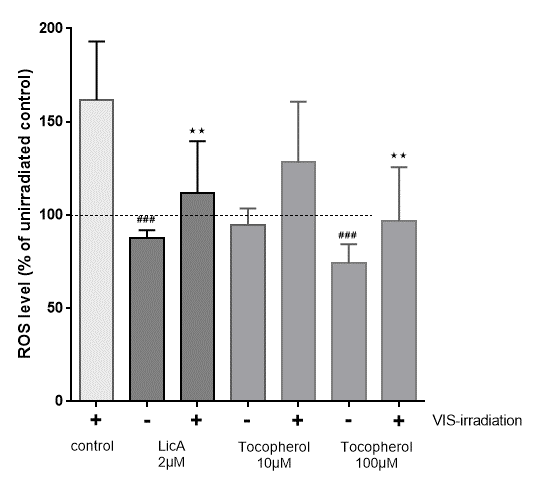


**Figure S9:** Effect of alpha-Tocopherol on VIS-induced ROS. The level of ROS in untreated fibroblasts was set to 100 %. Fibroblasts were incubated with LicA and alpha-Tocopherol for 24 hours. Thereafter, the cells were irradiated with 150 J/cm^2^ VIS and ROS levels were determined. Significant difference as marked, ^###^p≤ 0.001versus unirradiated control, **p≤ 0.01 versus irradiated control.
